# Outcomes of Brain Metastasis from Lung Cancer

**DOI:** 10.3390/cancers17020256

**Published:** 2025-01-14

**Authors:** James M. Mobley, Kerry I. Phillips, Quan Chen, Ellen Reusch, Niharika Reddy, Julia B. Magsam, Laurie E. McLouth, Bin Huang, John L. Villano

**Affiliations:** 1 Division of Medical Oncology, Department of Internal Medicine, University of Kentucky Markey Cancer Center, Lexington, KY 40536, USA; jamesmobley@uky.edu (J.M.M.); kerry.phillips@uky.edu (K.I.P.); ellen.reusch@uky.edu (E.R.); niharika.reddy@uky.edu (N.R.); jbmagsam@georgetowncollege.edu (J.B.M.); 2 Department of Radiation Oncology, Mayo Clinic, Scottsdale, AZ 85259, USA; qchen@abbvie.com; 3 Department of Behavioral Science, University of Kentucky, Lexington, KY 40536, USA; laurie.mclouth@uky.edu; 4 Kentucky Cancer Registry, University of Kentucky Markey Cancer Center, Lexington, KY 40536, USA; bhuan0@uky.edu

**Keywords:** lung cancer, brain metastasis, non-treatment, palliative care

## Abstract

**Background/Objectives**: Little information has been published on patients diagnosed with brain metastasis secondary to lung cancer. Correlating outcome patterns (hospice care, lost to follow-up, death before hospice care or treatment) and specific characteristics of treated and untreated patients may identify subsets of patients who may benefit from treatment. **Methods**: We evaluated data from the Kentucky Cancer Registry and identified 284 cases who were diagnosed with brain metastasis secondary to non-small cell lung cancer (NSCLC) between 1 August 2016, and 31 December 2019. We evaluated type and timing of treatment received, as well as focused on those patients who did not receive treatment. For those patients who did not receive treatment, various characteristics that may have impacted their decision or ability to undergo follow-up were also evaluated. This included social history, disease burden, as well as oncology treatment timelines. Lastly, due to the high smoking rate in Kentucky, we conducted an analysis of patient tobacco use. **Results**: Our results show that 61 cases (21.8%) never received treatment for lung cancer with brain metastasis. Further analysis of the non-treated cases demonstrated that 19 cases (31.1%) never met with an oncology team while in the hospital or after discharge; 14 of the 61 cases (23.0%) were too sick to receive treatment and died prior to having the option of treatment; and 47 of the 61 cases (77.0%) may have had the option of treatment but declined. **Conclusions**: Historically, patients with brain metastases have faced poor prognoses and limited treatment options. However, advancements in systemic chemoimmunotherapy and targeted therapies have introduced new treatment possibilities, offering improved symptom control and the potential for prolonged survival. This analysis is crucial for identifying potential barriers to care, optimizing resource allocation, and guiding future research.

## Simple Summary

Our study assessed lung cancer patient profiles and their outcomes in cases of brain metastasis due to lung cancer at a large academic medical center in the United States. We evaluated 284 cases who were diagnosed with brain metastasis secondary to lung cancer. We studied the type and timing of treatment received as well as focused on those patients who did not receive any treatment. For those patients who did not receive treatment, various characteristics that may have impacted their decision or ability to undergo follow-up were also evaluated. This included social history, disease burden, as well as oncology treatment timelines. Lastly, due to the high smoking rate in Kentucky, we conducted an analysis of the patient tobacco use as well. Our paper helps identify important patterns in this group of patients, which could help improve patient care.

## 1. Introduction

Lung cancer continues to be the leading cause of cancer mortality among both men and women across the United States, with more than 350 deaths occurring each day [1]. The United States cancer statistics data by the Centers for Disease Control and Prevention (CDC) show that Kentucky has the highest lung cancer incidence in the United States, with an age-adjusted incidence rate of 79.3 per 100,000 people per year in 2021. The number of lung and bronchus cancer cases reported was 4797. Kentucky was the state with the second highest mortality rate after West Virginia. It had a mortality rate of 47.7 per 100,000 people per year in 2022, with 2898 cancer deaths being reported [2]. Risk factors, such as smoking, are common in Kentucky, with over 25% of residents smoking. Additionally, many residents are at risk for exposure to radon, air pollution, and heavy metals [3]. Rural communities have a distinct genetic susceptibility, particularly in Appalachian Kentucky, where cancer disparities are observed [4].

Although there are extensive data on the treatment of various malignancies, limited investigations have been performed on the variables leading to non-treatment, particularly for a disease for which survival has recently improved. While some treatments remain palliative, refined surgical and radiation techniques have led to expansive options for patients who, previously, could only receive supportive care. Despite the advances in medicine, many patients still receive no treatment for their cancer, especially those with advanced disease. One study of 113,885 cases with invasive cancer found that 12.3% of all cases and up to 19% of the cases with lung/bronchial cancer did not receive treatment for their malignancies [5].

Unfortunately, many patients with lung cancer present with advanced disease. In particular, the incidence of brain metastases is increasing. This is due to prolonged survival stemming from more effective systemic treatments as well as improved detection of brain metastases on more accessible, higher resolution imaging [6,7,8,9]. When considering NSCLC, which is one of the most common malignancies causing brain metastases, observational studies have indicated that 30–43% of the patients develop isolated brain metastases without evidence of other metastatic disease [10,11]. In addition, approximately 30% of the patients with NSCLC have brain metastases at the time of diagnosis [12]. Small cell lung cancer also has a sufficiently increased risk of developing brain metastases, such that prophylactic cranial irradiation is often considered; approximately 20% of the patients present with brain metastases at diagnosis, and a sizeable proportion of those patients are asymptomatic [13,14]. While the mechanisms leading to the development of brain metastases in patients with lung cancer continue to be investigated, previous studies have suggested that variables such as EGFR mutation status, female gender, being a non-smoker, and young age are associated with a high risk of central nervous system (CNS) disease [15].

The prognosis for patients with brain metastases due to lung cancer has historically been noted to be poor, with a median overall survival of approximately 4.9 months for small cell lung cancer and 7.0 months for NSCLC [16]. However, therapeutic advances (such as immunotherapy and targeted therapies) have led to increased survival for advanced-stage lung cancer patients. These therapies have led to a more nuanced prognosis, even for those with brain metastases. For patients with EGFR/ALK gene alterations and brain metastases, for example, the median overall survival has been reported as high as 46 months [17]. Despite the improvements in treatment, many patients still receive no treatment for their cancer. One study of 113,885 patients with invasive cancer found that 12.3% of all patients and 19% of lung/bronchial cancer patients received no treatment [5]. The American Lung Association estimates that the national rate of non-treatment for patients diagnosed with lung cancer is 21.1% [18]. Reasons for non-treatment have not been well characterized, but likely include continued fatalism about the diagnosis, stigma surrounding lung cancer and its treatment [19], as well as social determinants such as distance to care, especially for patients with a history of tobacco use [20].

The purpose of this study was to examine the treatment patterns of advanced-stage lung cancer patients with brain metastases examined at a large NCI designated cancer center and to evaluate clinical and sociodemographic factors. Ultimately, these data will identify potential risk factors for non-treatment and help in performing a detailed analysis of the related potential causes. By uncovering patterns in the social histories and outcomes of patients with brain metastasis, we can better understand the obstacles that these individuals face, such as substance use, socioeconomic challenges, or healthcare system limitations. This understanding will allow for a more strategic allocation of resources, including targeted interventions and support services, and provide a foundation for research into improving treatment access, equity, and outcomes in similar populations.

## 2. Materials and Methods

Using the Kentucky Cancer Registry (KCR) Surveillance, Epidemiology, and End Results (SEER) program registry, a report on cases of brain metastases secondary to lung cancer diagnosed from 1 August 2016 to 31 December 2019 was compiled. Only cases seen or treated at the University of Kentucky (UK) were selected for this study. This was done to ensure that the records could be audited via chart review, and information on treatment history could be collected. The records of inpatient hospital admission at UK, treatment with radiation therapy, and neurosurgical services provided were found to be incomplete compared to the KCR data. In total, 334 cases were obtained, and 50 were excluded due to incomplete or poor follow-up information. The remaining 284 cases had verifiable courses of treatment available. Cases diagnosed or treated outside the UK for most of their treatment were still included for analysis if documentation of treatment history or outcome was available.

The time of diagnosis was included to determine if lung cancer and brain metastases were diagnosed concurrently or sequentially. The types of treatments received, including the percentage of cases that did not receive any treatment, were analyzed alongside tobacco use history in relation to the receipt of treatment (mixed tobacco products in terms of tobacco use status are defined by the KCR as the usage of any combination of tobacco products, including cigarettes, cigars, pipe tobacco, chewing tobacco, etc.). For the cases that did not receive any treatment, various characteristics that may have impacted their decision/ability to undergo follow-up were evaluated, such as social history, disease burden by M1b/c status, and oncology treatment timelines. Lastly, the distance (in miles) between the home address of each case and the UK was evaluated for all cases, treated cases, and non-treated cases.

## 3. Results

**Figure 1:**
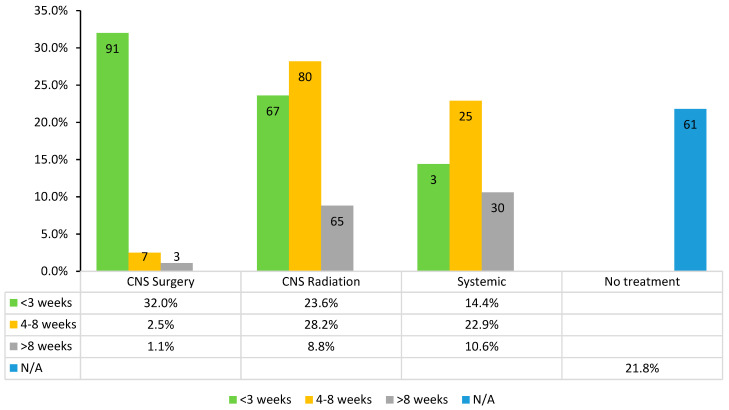
Timing and types of treatments received (the number of cases is shown inside the bars; the treatments were not mutually exclusive).

Of the 284 cases included in the study, 223 (78.5%) received anti-cancer treatment after diagnosis with brain metastases. A total of 136 cases (47.9%) received systemic treatment: 14.4% received their initial treatment within 3 weeks of brain metastases diagnosis, 22.9% received their initial treatment from 4 to 8 weeks after diagnosis, and 10.6% received treatment beyond 8 weeks after diagnosis. A total of 101 cases (35.6%) received CNS-specific surgical treatment after brain metastases diagnosis: 32.0% had surgery within 3 weeks, 2.5% had surgery from 4 to 8 weeks after diagnosis, and 1.1% had surgery beyond 8 weeks after diagnosis (Figure 1).

The role of focal vs. whole brain radiation treatment was also evaluated. Of the 172 cases that received CNS-specific radiation treatment, 97 (56.4%) received WBRT, 51 (29.7%) received focal radiation (gamma-knife, PBRT, or SBRT), and 24 (14.0%) received both.

Tobacco use was found to be the only statistically significant social factor associated with treatment decisions, with a *p*-value of 0.042. Of the 284 cases represented in this study, 257 (90.5%) were current cigarette smokers, 4 (1.4%) used mixed tobacco products, 13 (4.6%) never used tobacco, and 10 (3.5%) had an unknown history of tobacco use.

**Figure 2:**
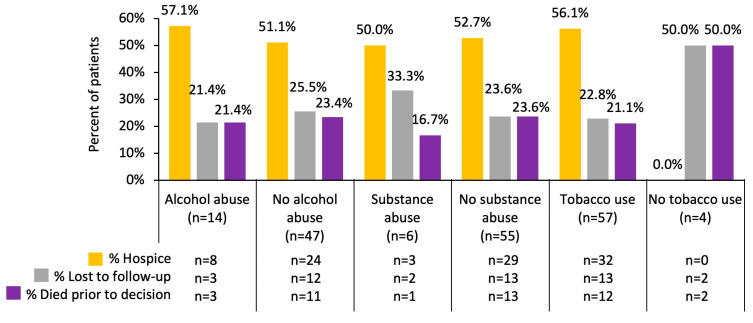
Outcomes of cases who received no treatment (*n* = 61) by social history (the number of cases is shown inside the bars; alcohol abuse *p* = 0.999, substance abuse *p* = 0.864, tobacco use *p* = 0.042).

The social histories of the 61 cases who received no treatment after their diagnosis of brain metastases secondary to lung cancer were evaluated; 14 (23.0%) had a history of alcohol abuse, 6 (9.8%) had a history of substance abuse, and 57 (93.4%) had a history of tobacco use. Additionally, 8 (57.1%) of the 14 cases with a history of alcohol abuse went to hospice, 3 (21.4%) were lost to follow-up, and the remaining 3 (21.4%) died prior to receiving any treatments or going to hospice. Of the six patients with a history of substance abuse, three (50.0%) went to hospice, two (33.3%) were lost to follow-up, and one (16.7%) died prior to receiving any treatment or going to hospice. Of the cases with a history of tobacco use, 32 (56.1%) went to hospice, 13 (22.8%) were lost to follow-up, and 12 (21.1%) died prior to receiving any treatments or having the option of going to hospice (Figure 2).

**Figure 3:**
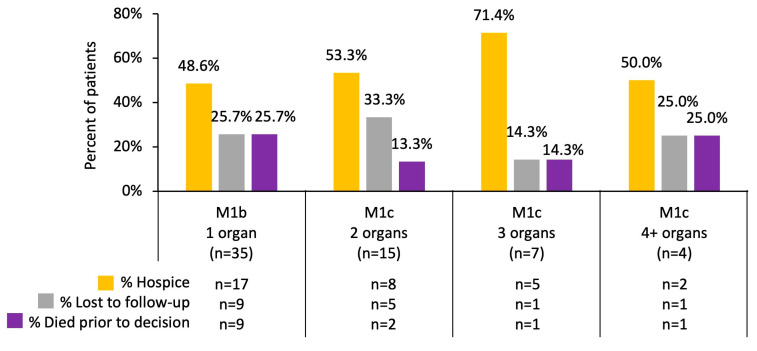
Outcomes of cases who received no treatment (*n* = 61) by disease burden (the number of cases is shown inside the bars) (*p* = 0.9337).

Of the 61 cases that received no treatment, 35 (57.4%) were labeled as M1b with one organ site involvement outside the lung/chest cavity (48.6% went to hospice, 25.7% were lost to follow-up, and 25.7% died before any treatment or the option of going to hospice), 15 (24.6%) cases were labeled as M1c with two organ site involvements outside the lung/chest cavity (53.3% went to hospice, 33.3% were lost to follow-up, and 13.3% died before any treatment or the option of going to hospice), 7 (11.5%) cases were labeled as M1c with three organ site involvements (71.4% went to hospice, 14.3% were lost to follow-up, and 14.3% died before any treatment or the option of going to hospice), and 4 (6.6%) cases were labeled as M1c with four or more organ site involvements (50.0% went to hospice, 25.0% were lost to follow-up, and 25.0% died before any treatment or the option of going to hospice) (Figure 3).

**Figure 4:**
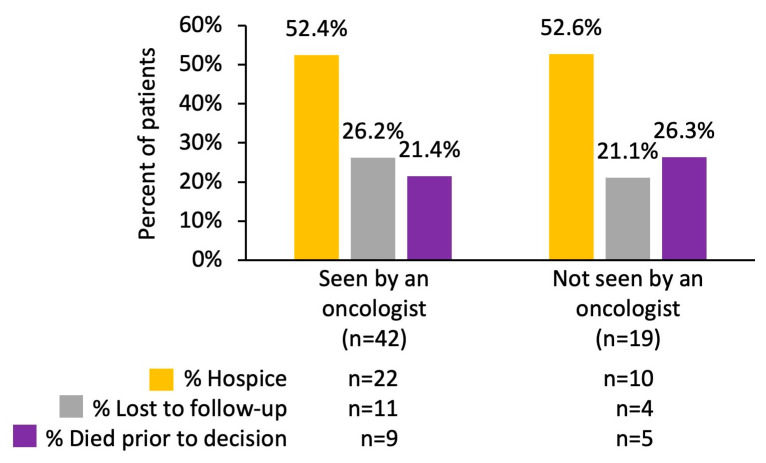
Outcomes of cases who received no treatment (*n* = 61) by an oncologist (the number of cases is shown inside the bars). About half of the patients in both groups entered hospice, while the remaining half were fairly evenly split between those lost to follow-up and those who died before a treatment decision was made.

In addition, 42 (68.9%) of the 61 cases that did not receive treatment were seen by oncologists either in-house at the time of diagnosis or after being released from the hospital. Of those 42 cases, 22 (52.4%) went to hospice, 11 (26.2%) were lost to follow-up, and 9 (21.4%) died prior to receiving any treatments or having the option of going to hospice. The remaining 19 (31.1%) of the non-treatment cases were not seen by an oncologist after their diagnosis: 10 (52.6%) went to hospice, 4 (21.1%) were lost to follow-up, and 5 (26.3%) died prior to receiving any treatment or having the option of going to hospice (Figure 4).

Further analysis demonstrated that while 14 of the 61 non-treatment cases were unable to receive treatment and died prior to having the option of treatment, the remaining 47 (77.0%) may have benefitted from treatment, as they were eligible for treatment based on the characteristics of their disease at the time of diagnosis, but they denied treatment.

The prognosis for most patients with advanced brain metastases is such that they are eligible for hospice care, which only accepts patients who have a 6-month or shorter prognosis. The time between the moment when patients were diagnosed with brain metastasis to that when they entered hospice care was evaluated as a measure of prognosis. Of the 61 non-treatment cases considered for the analyses (outlined in Figure 3 and Figure 4), 32 went to hospice. Of the 32 cases who went to hospice, 28 (87.5%) entered hospice less than 1 month after being diagnosed with brain metastasis secondary to lung cancer and 4 (12.5%) entered hospice between 1 and 6 months after being diagnosed with brain metastasis secondary to lung cancer. In contrast, 100 of the 284 patients who received treatment went to hospice, 41 (41.0%) of which entered hospice less than 1 month after their diagnosis with brain metastasis secondary to lung cancer, 41 (41.0%) entered hospice from 1 to 6 months after their diagnosis with brain metastasis secondary to lung cancer, 14 (14.0%) entered hospice 7–12 months after their diagnosis of brain metastasis secondary to lung cancer, and the last 4 (4.0%) entered hospice more than 12 months after their diagnosis with brain metastasis secondary to lung cancer.

Upon reviewing the disease burden, we found no difference in the distribution of disease burden in patients who sought treatment in comparison with those who did not receive treatment. Therefore, a higher disease burden does not seem to influence whether patients seek treatment for their diagnosis of brain metastasis secondary to lung cancer.

Finally, we analyzed how the distance between each patient’s home address and the UK impacted the likelihood that they did or did not receive treatment. Our data demonstrate that the distance between each patient’s home address and the UK did not make a difference in the likelihood of receiving treatment.

In summary, the individuals who did not seek treatment commonly exhibited certain characteristics, including tobacco use, M1b cancer status with one organ involvement outside of the lung or chest cavity, no prior history of an oncology visit, and a condition where treatment would likely have been beneficial. On the other hand, factors such as substance abuse, alcohol abuse, disease burden, and distance from the University of Kentucky did not appear to influence the treatment decisions.

## 4. Discussion

While the treatment of metastatic lung cancer, especially for patients with brain metastases, has advanced significantly, many patients still do not receive treatment. Unfortunately, the reasons for non-treatment have not been well characterized in this population. This analysis identified 47 cases (16.5% of all cases in the study) who may have been eligible for but did not receive treatment. This is within the context of care at a tertiary NCI-designated cancer center in the state having the highest incidence of and the second highest mortality rate for lung cancer in the United States [2]. The diverse backgrounds of UK lung cancer patients combined with the sheer number of cases treated annually make these results applicable to other centers; our findings span socioeconomic backgrounds, geographic location, and several other demographics.

Overall, tobacco was the most common chemical dependency seen among patients who did not receive treatment, with 93.4% of the patients having ongoing or previous tobacco use. While numerically slightly higher than that in the overall study population (90.5%), the frequency of smokers among the patients with brain metastases due to lung cancer in the KCR database is comparable to that reported in published literature [21]. While smoking may be common among patients with lung cancer and those with brain metastases due to it, the role of smoking status in determining whether a patient is likely to receive treatment is unclear. While well-published evidence shows that the smoking status of lung cancer patients influences outcomes [22,23,24,25], emerging data show an increase in brain metastasis velocity associated with increased smoking exposure [21]. Regardless, a survey among oncology providers on their practices regarding their advice on smoking cessation for patients with lung cancer demonstrated a hesitancy to encourage smoking cessation for patients with advanced disease despite over 98% of the same providers agreeing or strongly agreeing that quitting smoking benefits cancer patients [26]. This highlights the futility with which some providers still approach patients with advanced lung cancer, especially those with brain metastases. While this database review is unable to comment specifically on individual patient treatment decisions, it is worth exploring whether the smoking status influences the oncologist choice of offering treatment to patients with advanced malignancies, especially lung cancer.

While patients presenting with an increased volume of metastatic disease are expected to have a worse prognosis and, thus, may not receive treatment, 57.4% of the non-treated patients in this study had an M1b disease burden (metastasis to one site outside of the primary site). Because this study only included patients with brain metastases due to lung cancer, these 35 patients had no other metastatic sites. While the prognosis of brain metastases due to lung cancer is historically poor, there are now numerous local and systemic treatment options for the palliation of symptoms and, potentially, disease control. In fact, less than half (47.9%) of the patients in this study received systemic treatment at all for their metastatic lung cancer. As cancer care becomes more personalized with the increased use of tumor sequencing and targeted therapy, the management of metastatic disease becomes more nuanced. However, it should be noted that the newest treatments were not available at the time that some of the patients were monitored. The literature regarding systemic and targeted therapy for patients with brain metastases due to lung cancer continues to advance, giving patients with oligometastatic disease even more options regarding their care [27,28,29,30]. This underscores the importance of patient and provider education regarding the rapidly evolving treatment options for patients with metastatic lung cancer.

Oncology care providers can address the identified barriers to treatment through several specific strategies such as offering direct transportation services like ridesharing or insurance-provided transportation; implementing tele-oncology services to improve access to cancer specialists, especially for rural patients; developing support groups or peer mentoring programs for patients; and simplifying paperwork and reducing the number of clinic visits when possible [31,32].

Our study also identified many patients who were not evaluated by an oncology provider. Nearly one-third (31.1%) of the patients who did not receive treatment were never seen by an oncologist. Even if this number includes all 14 of the patients who were too ill to receive treatment, there were still patients who potentially may have been able to receive treatment but never met with a provider who could offer such treatment. This study only included patients evaluated at a large academic center at which oncology services are highly available and well established. This information raises concern for patients in underserved areas, where oncologist availability is scarce. This aspect of patient care was evaluated by examining the likelihood of patients receiving treatment based on their home address’s distance from the reporting medical center. There was no correlation between treatment administration and the distance between the medical center and the patient home address. While there is often concern that patients will not want to travel long distances for their cancer care or that they are unable to receive care close to their home, our study found that even patients with a home address more than 100 miles away were just as likely to receive treatment as they were to receive no treatment. This also could limit an evaluation of the practices at rural centers in the state, but this is an area of interest for future research.

As a retrospective study, this study had limited ability to assess the rationale behind each individual patient treatment plan. We were also unable to examine patient-centric factors and patient preferences regarding their willingness to receive treatment. Although we were able to review documented cases of treatment refusal, more valuable information may have been obtained from a conversation with the patient and care team. Additionally, the size of the population included in the study is fairly small. Our findings may have been influenced by the practice environment being a large, academic center with widely available oncology and subspecialty care. While this might have biased our results, it would be expected to increase the proportion of patients referred for treatment, although we were unable to directly compare the number of the studied patients with that of patients treated at other centers in this study.

## 5. Conclusions

In conclusion, although this patient population has historically experienced poor prognosis and limited treatment options, advances in radiation modalities and surgical techniques combined with advances in systemic chemoimmunotherapy and targeted therapies have opened new treatment options that could offer improved symptom control with the possibility of prolonged survival. Our investigation provides preliminary insights into potential patterns within this patient group, which may contribute to improving patient care. Additional research into provider and patient perceptions as well as broader research in community healthcare settings may reveal additional patients who could benefit from treatment options that may not have been available in the past.

### Limitations

In our study, out of the 334 cases obtained, 50 were excluded due to incomplete or inadequate follow-up information. This rigorous selection process was essential to ensure the integrity and reliability of our analysis. We focused on the remaining 284 cases for an in-depth analysis, as they provided a verifiable course of treatment.

We did not collect qualitative data on the reasons why patients refused treatment or the role of healthcare providers in influencing these decisions. Such insights could have provided a more comprehensive understanding of patient behaviors and attitudes toward treatment options. Future research should consider qualitative interviews or surveys to obtain more detailed insights into these aspects, thereby enhancing our depth of understanding and potentially informing improved patient-centered care strategies.

Our paper is important as it addresses the challenges of lung cancer care, particularly for patients with brain metastases, in a region that experiences one of the highest incidences of lung cancer in the country. As the only NCI-designated comprehensive cancer center in Kentucky, we offer vast resources to our many patients. It is likely that smaller facilities may not provide the same level of care. Even with our extensive experience and care for this population, we are not always able to provide optimal care.

We believe our experiences are generalizable and can be informative for other facilities with less experience and resources for treating these patients. This is a single-institution study and, as such, has the potential downfalls of a homogenous population [33] and an institutional bias with respect to practices and protocols. However, we serve a large, diverse patient population. Our patient demographics are comparable to the national demographics in gender and race [34,35]. By examining our experiences and outcomes, we offer valuable insights that can help bridge the gap between specialized centers and smaller facilities with limited resources. This is important, as advances in chemoimmunotherapy and supportive care can significantly improve patient survival outcomes.

Our study provides insight on the challenges faced in Kentucky, and this can improve targeted interventions and policies aimed at enhancing cancer care across the region. Our findings contribute to a broader understanding of how to optimize treatment for lung cancer patients with brain metastasis, particularly those with complex needs, thereby advancing efforts to improve their survival outcomes and reduce disparities in healthcare access.

## Data Availability

The data presented in this study are available in this article.
